# Staphylococcal Pericarditis Causing Pericardial Tamponade and Concurrent Empyema

**DOI:** 10.1155/2019/3701576

**Published:** 2019-07-18

**Authors:** Divya Kondapi, Danny Markabawi, Andrew Chu, Harvir Singh Gambhir

**Affiliations:** Suny Upstate Medical University, 750 East Adams St., Syracuse, NY 13210, USA

## Abstract

Bacterial pericarditis is a rare presentation and is usually due to secondary infection from a hematogenous cause or can occur secondary to trauma, intrathoracic surgery, or due to spread of infection from a contiguous focus via ligaments that anchor the pericardium to its surrounding structures. Its course is fulminant characterized by a high mortality rate from sepsis, tamponade, and constriction. We describe a rare case of *Staphylococcus aureus* pericarditis with concurrent unilateral empyema. The patient rapidly developed tamponade and was successfully treated with antibiotics and urgent percutaneous pericardial drainage with placement of a temporary catheter. Treatment for bacterial pericarditis typically is 4–6 weeks long. Thoracic surgery should be consulted as soon as possible to determine need for surgical intervention, as fibrin deposition may occur, making percutaneous drainage incomplete and leading to complications of persistent purulent pericarditis or constrictive pericarditis.

## 1. Introduction

Acute bacterial pericarditis is rarely encountered in the modern antibiotic era [1]. Purulent pericarditis is a very serious form of bacterial pericarditis and is defined by the presence of frank pus in the pericardium [2]. It carries a high mortality rate as it can rapidly progress to tamponade and death. Bacterial involvement of the pericardium is usually due to contiguous or hematogenous spread and is very rarely seen without evidence of infection elsewhere in the body [3]. Here, we present a severe case of purulent pericarditis with cardiac tamponade and empyema which was managed successfully with pericardial draining and intravenous antibiotics.

## 2. Case

We present a case of a 68-year-old female who is a smoker, with moderate aortic stenosis and a complex surgical history which included but not limited to a gastric bypass surgery 8 years prior to presentation and three prior joint arthroplasties. Her right shoulder and left hip replacement were performed about 1 year prior to her presentation, and her left knee arthroplasty was done about 18 years ago.

She was in her usual state of health until a week prior to seeking care. She presented to an outside hospital with a dry cough, shortness of breath, pleuritic chest pain, and fatigue. She denied fevers, abdominal pain, nausea, vomiting, dysuria, and diarrhea. She denied any history of intravenous drug use, and a urine screen for drugs of abuse was never performed. Her workup and cardiac imaging at the outside hospital revealed a large pericardial effusion, and she was thus transferred for a thoracic surgery evaluation for a pericardial window.

In our emergency department, she was found to be in atrial fibrillation with a heart rate between 140–160 bpm. She was afebrile at the time of presentation, and her initial blood pressure was 119/80 mmHg. She appeared cachectic and was breathing at 25 min.

Cardiovascular and respiratory examinations were significant for a pericardial rub and reduced breath sounds in the right lower lung base. Pulsus paradoxus was not appreciated, and her jugular venous pressure was normal.

**Figure 1:**
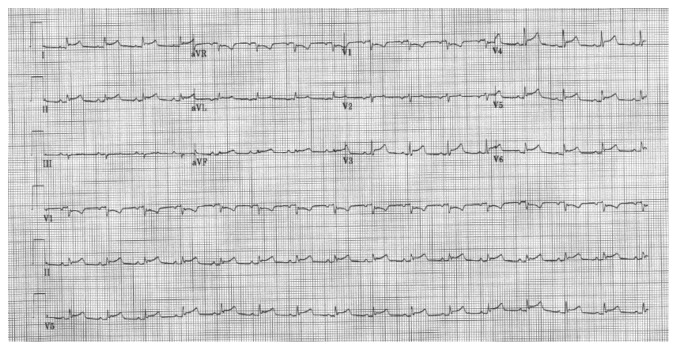
EKG demonstrating diffuse ST-segment elevation.

**Figure 2:**
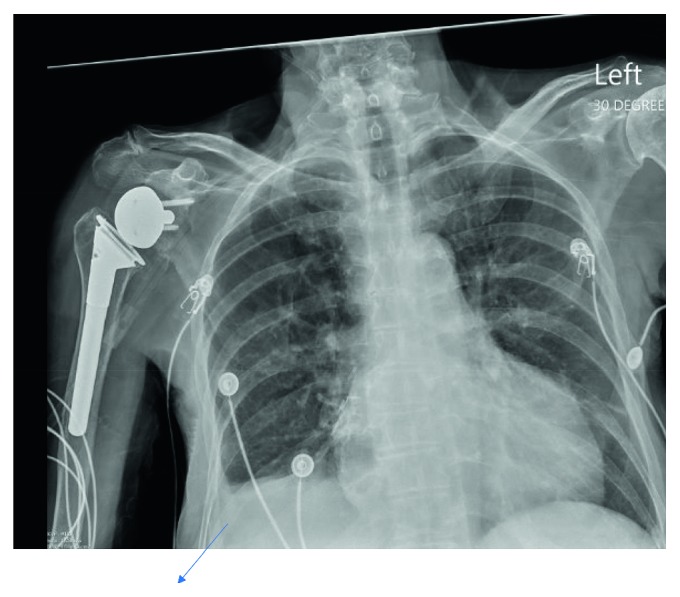
Enlarging right-sided pleural effusion that was later drained and found to be an empyema.

Her labs revealed leukocytosis of 16.6 K with 89% neutrophils, an ESR of 59 mm/hr, and a CRP of 174 mg/L. She had elevated transaminases with an ALT/AST of 391/698 U/L and INR of 2.19. Her EKG showed diffuse ST elevations consistent with pericarditis (Figure 1). Chest X-ray revealed a small right-sided pleural effusion and an enlarged cardiac silhouette (Figure 2). Bedside transthoracic echocardiogram was repeated and showed a large circumferential pericardial effusion with no evidence of tamponade. She was given a diagnosis of viral pericarditis leading to pericardial effusion and consequent hepatic congestion and coagulopathy. Thoracic surgery was consulted for the need to place a pericardial window but they felt it was not indicated at the time as she was not in tamponade at the time. However, within 2 hours, she had worsening tachycardia with a heart rate of 170 bpm and her blood pressure began to decline rapidly. Fluid resuscitation was initiated, her INR was reversed, and emergent pericardiocentesis was performed for concern of progression to tamponade. A percutaneous drain was inserted, and this drained over 350 mL of purulent fluid. The pericardial fluid had 627 WBCs with neutrophilic predominance, and cultures grew methicillin-sensitive *Staphylococcus aureus* (MSSA). Cytology was negative for malignancy. She was placed on IV vancomycin until final sensitivities resulted and oral colchicine. Blood cultures from the outside hospital came back positive for MSSA, and her antibiotic regimen was changed to IV oxacillin. She was monitored in the cardiac ICU. Transaminases and INR slowly improved with pericardial fluid drainage. During the hospital stay, a repeat chest X-ray revealed enlarging right- sided pleural effusion. While it could have been attributed to the large amounts of IV fluids she received earlier, it was decided that a thoracentesis be performed to rule out empyema. Pleural fluid was purulent as well, and analysis showed 790 WBCs with 85% neutrophils, a pH of 7.44, and glucose of 191 mg/deciliter. The pleural LDH was 178 mg/deciliter and protein content was 2.3 mg/dl, while the serum LDH was 267 mg/dl and serum protein was 5.3 mg/deciliter, meeting Light's criteria for exudative fluid. While the pH, protein ratio, and glucose were not suggestive of empyema, fluid grew MSSA as well. Computerized tomography of chest was done after the drainage, and it did not show a lung infiltrate. A right-sided chest tube was inserted. A transesophageal echocardiogram did not show any vegetation, and repeat blood cultures were negative on day 2. Given the fact that she had serositis involving multiple sites, autoimmune workup was performed, and it was negative. Orthopedics was also consulted given her history of multiple prostheses and the possibility of these becoming infected or even being the primary source. X-ray imaging did not reveal any loosening of hardware, and the patient did not have any symptoms concerning for septic arthritis. Her blood cultures continued to remain negative. Therefore, no surgical intervention was pursued.

Her pericardial drain and chest tube were eventually removed, and she was discharged on IV cefazolin for 6 weeks and oral colchicine with a follow-up at the infectious disease clinic.

## 3. Discussion

The pericardium and its fluid provide lubrication for the moving surfaces of the heart and also create a barrier for the spread of infection. Acute pericarditis is an inflammatory process involving the pericardium. The cause of acute pericarditis is usually viral or idiopathic in 90% of the cases. Bacterial pericarditis only constitutes 1-2% of these cases in the post-antibiotic era [4]. The organisms most commonly known to cause purulent pericarditis are *Staphylococcus*, *Streptococcus*, *Hemophilus*, and *M. tuberculosis*. *Mycobacterium avium* intracellulare pericarditis can occur in higher proportions in those with AIDS [5].

Purulent pericarditis occurs either due to hematogenous spread or by direct spread. Since the pericardium has ligamentous attachments to the sternum, vertebral column, diaphragm, pleura, and anterior mediastinum, infection can track along these ligaments. The contiguous spread from the lung or pleura via the pleuropericardial ligaments accounts for most of these cases [6]. Risk factors for bacterial pericarditis include immunosuppression, cardiothoracic surgery, trauma, preexisting catheters in the pericardial cavity, or preexisting pericardial effusion [7].

Differentiating between viral and bacterial pericarditis based on clinical presentation and imaging poses a diagnostic challenge. In one retrospective review, a large number of patients with bacterial pericarditis presented with signs of infection such as fever and chills. Chest pain was seen in about 25 to 37% of the patients. The physical examination findings of pericardial rub and pulsus paradoxus were found in less than 50% of the cases of pericarditis and pericardial effusion [6]. Laboratory studies may show evidence of systemic inflammation such as leukocytosis with neutrophilia in the case of bacterial pericarditis. Elevated CRP and ESR can also be appreciated. Radiographic findings may show pleural effusions, abnormal cardiac silhouette, and widened mediastinum. EKG findings that are consistent with the findings of pericarditis or pericardial effusion are diffuse ST-segment elevation, PR-segment depression, low voltage, or electrical alternans. Echocardiogram can show evidence of increased pericardial fluid [5]. However, it is difficult to differentiate purulent pericarditis from other causes of pericarditis or pericardial effusion with echocardiogram alone. Strong clinical suspicion is needed to make prompt diagnosis as early aggressive treatment is required for this near-fatal disease.

Although drainage of the pericardial fluid is required for adequate source control and, in some cases, achieving hemodynamic stability, this should not delay initiation of antibiotic therapy. Empiric treatment involves an antistaphylococcal agent, with a 3rd generation cephalosporin and fluoroquinolone [5].

The Colchicine for acute Pericarditis (COPE) and Colchicine for Recurrent pericarditis (CORE) trials were the first prospective, open-label, randomized studies to support the use of colchicine for acute and recurrent pericarditis [8, 9]. However, patients with neoplastic or bacterial pericarditis were excluded from these studies. Further studies are needed to clarify the role of colchicine in bacterial pericarditis.

Pericardial fluid drainage is always indicated when the patient presents with pericardial tamponade and when there is suspicion for purulent or malignant pericardial effusion. Aggressive management is necessary as purulent pericarditis carries a high mortality risk. The choice of pericardial drainage method appears to be controversial. The various surgical modalities for evacuation of bacterial pericarditis include subxiphoid drainage, fibrinolysis, wide bore pericardiotomy, pericardial window, and lastly pericardiectomy which could be partial or total [10, 11]. Simple percutaneous drainage alone is usually insufficient and could cause the disease process to evolve into a constrictive or persistent form of purulent pericarditis because of loculations and adhesions [12–14]. Fibrin formation increases during the first week of the disease, and fibrosis may appear after 2 weeks [10]. Early invasive surgical management is thus recommended to avoid these complications. This is also supported by a retrospective series, in which partial pericardiectomy and total pericardiectomy were associated with lower mortality compared to simple drainage alone [6, 10, 12, 14]. Patients in these case series however had pericardial fluid with exudative loculations, extensive granulation tissue, and septate adhesions [12], which our patient did not have. Pericardiectomy is not with potential serious complications; hence, less-invasive modalities are attempted if the disease process is in its early stages.

The concern of pericardial lavage with saline and antibiotics, or povidone iodine alone is that it cannot dissolve thick adhesions [10, 15, 16]. However, intrapericardial fibrinolysis with streptokinase and streptodornase may be helpful in these cases. This requires frequent irrigation of the pericardial cavity with the above agents using large catheters, which liquifies the exudate. Streptokinase acts by dissolving blood clots and fibrinous exudate, and streptodornase liquefies the nucleoprotein of pus [17, 18]. Upon liquefaction, penetration to antibiotics can also be enhanced. Primary fibrinolysis is performed immediately after drain insertion, and rescue fibrinolysis is used if there is recurrence or incomplete drainage. However, thick loculations cannot be liquefied by a fibrinolytic agent alone [19]. These agents can only dissolve fibrin but have no effect on fibrosis. Theoretical complications of fibrinolysis include hemorrhagic transformation of PP [10], which is more a consequence of PP itself rather than local fibrinolysis, and the complication of tamponade based on the instillation volume [20], but this risk is lowered if the volume of the fibrinolytic agent is less than the amount of pericardial fluid that is drained just prior to its instillation.

Classical subxiphoid pericardiotomy has the advantage of achieving more permanent and complete drainage than pericardiocentesis while avoiding the risk of sternal or pleural contamination and is useful in the critically ill patients where a thoracotomy may be too aggressive. Furthermore, it allows for mechanical breakdown of loculations and septations by the surgeon. The disadvantage with this approach is difficult or lack of access to posterior fluid loculations, which would then require pericardioscopy to facilitate the drainage of the same [21, 22].

An external pericardial window done by median sternotomy helps with pericardial exposure and easier removal of adhesions and loculated fluid but carries the risk of sternal contamination. A pleuropericardial window on the other hand performed by either video-assisted thoracic surgery (VATS) or anterior thoracotomy carries the risk of pleural cavity contamination and would therefore not be recommended in our patient [22].

In patients with high fibrin content in the pericardial fluid, tendency for local fibrosis, recurrent tamponade, persistent infection, or progression to constrictive pericarditis, pericardiectomy is indicated. It is performed through an anterolateral thoracic approach or a median sternotomy. This procedure carries more morbidity and mortality risk compared to pericardiotomy, partly due to the fact that patients in whom it is indicated are more likely to have multiple complications and signs of hemodynamic instability. However, it usually allows for complete drainage of the pericardial fluid [23, 24].

Causes of death in those with purulent pericarditis are tamponade, sepsis, or constriction [5, 6]. Our patient had MSSA bacteremia from an unclear source, with empyema and bacterial pericarditis. The coexistence of these conditions could be explained by tracking of the infection along the pleuropericardial ligament. It is also possible that the bacteria originally seeded the pericardium by hematogenous spread from another unclear source. We did explore the possibility of our patient having serositis from connective tissue disease. Autoimmune workup was sent and was negative. Our patient was treated successfully with IV antistaphylococcal agents and a percutaneous pericardial drain. The pericardial drain was removed once it stopped draining, and a 2D echo showed trivial effusion with no evidence of loculation. CT thorax done 6 weeks after starting antibiotics showed complete resolution of both pleural and pericardial effusions, with no evidence of pericardial thickening or adhesions.
